# Pentraxin 3: An Immuno-Regulator in the Lungs

**DOI:** 10.3389/fimmu.2013.00127

**Published:** 2013-05-31

**Authors:** Jyoti Balhara, Latifa Koussih, Jingbo Zhang, Abdelilah Soussi Gounni

**Affiliations:** ^1^Department of Immunology, University of Manitoba, Winnipeg, MB, Canada; ^2^University Saint Boniface, Winnipeg, MB, Canada; ^3^Xinqiao Hospital, Third Military Medical University, Chongqing, China

**Keywords:** PTX3, TNF, IL-1β, immune system, complements, lungs

## Abstract

Pentraxin 3 (PTX3) is a soluble pattern recognition receptor that is a humoral component of the innate immune system. It interacts with pathogenic moieties, infected and dying host cells and facilitates their removal through activation of appropriate innate and adaptive mechanisms. PTX3 is secreted by a diverse variety of cells, ranging from immune cells to structural cells, in response to Toll like receptor (TLR) engagement, inflammatory stimuli, and physical and chemical stress. Further, PTX3 plays an essential role in female fertility as it facilitates the organization of extracellular matrix in the cumulus oophorus. Such activity is also implicated in post-inflammation tissue repair. PTX3 is a multifunctional protein and plays a non-redundant role in providing immunity against potential immunological dangers. Thus, we assessed its role in lung immunity, as lungs are at a constant risk of infections and tissue damage that is attributable to perpetual exposure to foreign agents.

## Introduction

Pentraxins are a superfamily of evolutionarily conserved, specific pattern recognition proteins that play a salient role in the innate immune system. Based on size, these multifunctional proteins are divided into long and short pentraxins. Long pentraxins include the prototypic pentraxin 3 (PTX3), the recently identified PTX4, and neuronal pentraxins 1 (NP1) and NP2 whereas short pentraxins consist of C-reactive protein (CRP) and serum amyloid P (SAP) (Gewurz et al., 1995; Garlanda et al., 2005).

Phylogenetic analysis has demonstrated conservation of short and long pentraxins in human, mouse, rat, opossum, chicken, and some lower vertebrates (Martinez de la Torre et al., 2010). Although all pentraxins evolve from a common ancestor, PTX3 alone forms a separate cluster and seems to originate directly from the common ancestral pentraxin very early in evolution (Martinez de la Torre et al., 2010).

Pentraxin 3 has been identified as biomarker of several immunopathological states and its relevance with the resolution of infections and diseases has also been studied. In this review, we detail the general structure, expression, and functions of PTX3. Also discussed are current findings, which suggest an important role of PTX3 in immunological states, particularly those that are associated with the lungs.

## PTX3 Gene Structure

**Figure 1:**
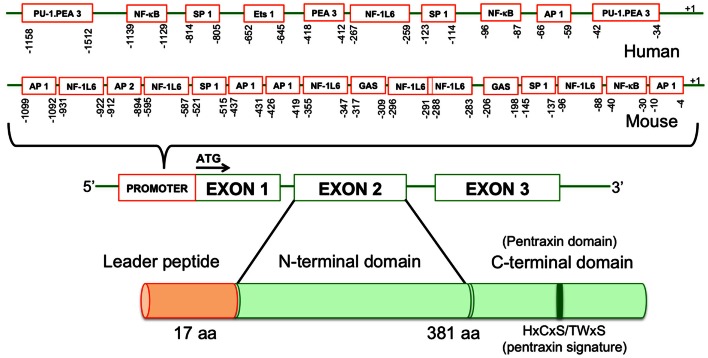
**Molecular structure of PTX3 gene in human and mouse**. PTX3 gene is organized into promoter region and three exons: the first exon encodes for leader peptide (17 amino acids) while the second and the third exons encode for N- and C-terminal domains of the protein (381 amino acids). Promoter region contains multiple transcription binding sites.

The human and murine PTX3 gene is localized on chromosome 3 (q22–28) (Breviario et al., 1992). The PTX3 gene is organized into three exons: the first and the second exons translate to the leader peptide and N-terminal domain, respectively, whereas the third exon which corresponds to second exon of short pentraxins, encodes pentraxin domain (Breviario et al., 1992) (Figure 1). The human and mouse PTX3 displays 92% amino acid conservation and 82% of these amino acid residues are identical (Introna et al., 1996; Garlanda et al., 2005). Significant homology between human and mouse PTX3 suggests that murine studies can be extrapolated to address human issues.

The human PTX3 promoter contains several potential cis acting elements including an NF-kB site, binding sites for activator protein 1 (AP-1), AP-2, specificity protein (Sp1), and gamma interferon activation site (GAS) (Altmeyer et al., 1995) (Figure 1). In addition to these elements, the murine PTX3 gene promoter sequence also contains binding sites for homeodomain (Hox)-1.3, and transcription factors belonging to Ets family. The mouse PTX3 gene contains a 44-bp stretch of alternating CA residues in its promoter region, which acts as an enhancer element. The murine promoter region also contains multiple NF-IL-6 binding sites while its human counterpart contain only one, which draws particular attention on the role of NF-IL-6 in the regulation of PTX3 expression (Darnell et al., 1994).

Unlike short pentraxins, the PTX3 promoter does not contain a consensus site for hepatic nuclear factor-1 (HNF-1), which accounts for the absence of its induction in liver (Darnell et al., 1994). Although human and murine PTX3 protein is homologous, the human promoter contains lesser transcriptional elements than does the murine promoter, an effect that needs to be studied using molecular and phylogenetic analysis.

## PTX3 Protein Structure

The name pentraxin is derived from the molecule’s pentagonal structure, which consists of five subunits. It was initially thought that PTX3 acquired a quaternary structure that is similar to short pentraxins, which consists of five identical 23 kDa subunits held together by noncovalent non-covalent interactions. However, the amino acid residues at the protomer interface that are required for pentameric structural formation in CRP, are not present in PTX3 (Introna et al., 1996). PTX3, exists mainly as octamers made from two tetramers and each subunit is held together by covalent bonds (Bottazzi et al., 1997; Garlanda et al., 2002; Inforzato et al., 2008). PTX3 octamers show greater functional activity as compared to tetrameric oligomers (Inforzato et al., 2008).

The PTX3 protein structure, like that of other pentraxins includes a pentraxin domain containing HxCxS/TWxS (where x is any amino acid) at C terminus (Figure 1). Its C-terminal domain consists of 203 amino acids, 57% of which are conserved in the entire pentraxin superfamily and the unique N-terminal domain consists of 178 amino acids (Agrawal et al., 2009). The PTX3 C- and N-terminal domains evolved independently, which explains why the C-terminal domain is widely conserved while the N-terminal domain is not (Martinez de la Torre et al., 2010). Mature PTX3 protein is approximately 40 kDa, but glycosylation at Asn220 increases the molecular weight to 45 kDa (Garlanda et al., 2005). Oligosaccharide moieties at Asn220 can be complex or heterogenous sialic and fucosyl sugar moieties. Unique glycosylation patterns are associated with different inflammatory cells and inflammatory stimuli that induce PTX3 production (Inforzato et al., 2006). Desialylation of PTX3 results in stronger binding to C1q and activates the classical complement pathway to a sizable extent (Inforzato et al., 2006) (Table 1). The PTX3 protein has two conserved cysteine residues at the C-terminus (Cys210 and Cys271) and seven other cysteine residues, which are exclusive to PTX3: three at the N-terminus (Cys47, 49, and 103) and four in the C-terminal region (Cys179, 317, 318, and 357). These cysteine residues form a network of disulfide bonds that maintains the PTX3 oligomeric structure. Interchain Cys47–Cys47 and Cys49–Cys49 bonds form dimers and these dimers are further held together by Cys103–Cys103 bonds that maintain tetrameric structures (Doni et al., 2006). These tetrameric structures are further assembled into octamers by disulfide bonds between Cys317 and Cys318, which are located on exposed loops of different protomers (Inforzato et al., 2008). The PTX3 C-terminal domain is formed by two antiparallel β sheets, stabilized by Cys210 and 271 and organized into a β-jelly roll topology (Pepys and Baltz, 1983; Goodman et al., 1996). Cys179 and 357 also form disulfide bonds and seem to limit the flexibility of the N- and C-terminal regions by linking them together (Inforzato et al., 2008). The N-terminal region, however, is predicted to assume a coiled coil conformation as it is made up of four alpha helices (Presta et al., 2007).

**Table 1:** **Pentraxin 3 interacts with various ligands to mediate diverse function**.

	Ligand	Role	Characteristics of interaction	Reference
1	P-selectin	Inhibition of neutrophil migration	C-terminal domain is required	Deban et al. (2010)
			Dependent on glycosylation pattern	
2	C1q	Complement activation	C-terminal domain of PTX3 is required	Inforzato et al. (2006), Baruah et al. (2006)
			Desialylation of PTX3 favors interaction
			Interaction is mediated through globular head of C1q	
			Inhibitory effect when C1q is in fluid phase	
			Calcium independent	
3	Ficolins	Complement activation	Calcium dependent	Ma et al. (2009), Gout et al. (2011)
			Interaction mediates through fibrinogen-like domain of ficolins	
			Glycosylation pattern in C-terminal domain of PTX3 is a determining factor	
4	Factor H	Complement activation	N- and C-terminal domains are required for interaction	Deban et al. (2008)
			Interaction is calcium dependent
			Glycosylation of PTX3 stabilizes the interaction	
5	MBL	Complement activation	Mediated through collagen like domain of MBL	Martin et al. (2009)
			Interaction dependent on calcium
6	FyRs	Opsonophagocytosis		Moalli et al. (2010), Lu et al. (2008)
7	FGF-2/8b	Anti-angiogenesis	Interaction through N-terminal domain (FGF-2)	Moalli et al. (2010), Lu et al. (2008), Rusnati et al. (2004), Camozzi et al. (2005, 2006)
8	TSG-6	Extracellular matrix deposition	Interaction through N-terminal domain	Salustri et al. (2004), Maina et al. (2009)
9	Galactomannan (component of conidia)	Disposal of conidia		Garlanda et al. (2002)
10	OmpA (*Klebsiella pneumoniae*)	Activation of immune response against *Klebsiella* (complement activation)	High affinity interaction Calcium dependent	Cotena et al. (2007), Jeannin et al. (2005)
11	Hemagglutinin	Inhibition of viral attachment to host cells	Calcium independent Sialic moiety on PTX3 and mannose on hemagglutinin are involved in interaction	Bozza et al. (2006), Reading et al. (2008)

*This table shows list of PTX3 ligands and role and characteristic features of their interaction*.

Recently, Delneste and coworkers showed that the PTX3 N-terminal domain is susceptible to cleavage by proteases, particularly neutrophil elastase and *Aspergillus fumigatus* proteases (Hamon et al., 2013). The serine protease inhibitor PMSF, antipain, and chymostatin were shown to inhibit *A. fumigatus* proteases activity but its activity was unaffected by aspartic-, metallo-, cysteine-, and aminopeptidase protease inhibitors (Hamon et al., 2013). Proteolytic cleavage of PTX3 has added an interesting aspect to the regulation of PTX3 expression and function and a detailed analysis is necessary to validate this phenomenon.

## Cellular Sources of PTX3

There is a growing body of evidence suggesting that PTX3 can be produced by many cell types and induced by various different stimuli (Breviario et al., 1992; Lee et al., 1993). It is due to this reason that PTX3 is capable to serve multiple functions depending upon condition. It is interesting to note that regardless of the source of its production (immune cells or structural cells), PTX3 plays a critical role in regulation of the humoral arm of innate immunity (Lee et al., 1993).

### Immune cells

Lymphoid cells such as T cells, B cells, and NK cells do not express PTX3. This highlights the significance of PTX3’s control on the innate immune system (Deban et al., 2011). However, the action of PTX3 is not limited to the innate immune system: PTX3 coordinates with adaptive immune system and facilitates protection against infections.

#### Dendritic cells

Among cells of immune system, myeloid cells, and especially dendritic cells (DCs), are the main source of PTX3 (Introna et al., 1996). An intricate network, as demonstrated by Doni et al. (2006) regulates its expression in myeloid DCs upon stimulation with the Toll like receptor (TLR) ligands, CD40L, IL-10, and IL-1β. However, no such effect was observed in plasmacytoid DCs. Macrophages also express PTX3. Macrophages from PTX3 overexpressing mice show an augmented phagocytic response to zymosan and *Paracoccidioides brasiliensis* (Deban et al., 2011).

#### Neutrophils

Neutrophils are the only granular cells reported to release preformed PTX3 in response to TLR agonists and microorganisms. PTX3 exists as a monomer in “ready to release” myeloperoxidase (MPO) negative granules containing lactoferrin and lactoferrin/gelatinase and assembles into multimers upon release. When neutrophils are activated in response to inflammatory stimulation, they release 25% of their PTX3. Part of the released PTX3 remains associated with neutrophil extracellular traps (NETs), which interacts with certain components of NETs (Jaillon et al., 2007; Daigo et al., 2012). Neutrophils are among the first cells to defend against foreign pathogens and the immediate release of PTX3 by these cells may be indicative of its importance in innate immunity.

### Structural cells and other cells

#### Adipocytes

Pentraxin 3 is induced by TNF in adipocytes (Abderrahim-Ferkoune et al., 2003). Preadipocytes also showed PTX3 expression, which decreases upon differentiation to adipocytes. In light of differential PTX3 expression in different differentiation stages of adipocytes, function of PTX3 in this process was found irrelevant. Additionally, a greater level of PTX3 mRNA was observed in adipose tissue of obese and obese diabetic mice as compared to WT mice. Although authors suggested this expression resulted from adipocytes, examination of cell-specific PTX3 production in these tissues is requisite (Abderrahim-Ferkoune et al., 2003). Altogether, more studies are required to determine the functional outcome of the role of PTX3 during differentiation and also in obese condition.

#### Cardiomyocytes

Pentraxin 3 is constitutively expressed in the human heart by cardiomyocytes. (Peri et al., 2000). However dying and necrotic cells release it in large quantities, contributing to its increased level in the blood of patients with acute myocardial infarction (AMI) (Peri et al., 2000). Although its exact role in healthy myocytes is not well understood, it is generally used as an indicator of tissue damage in AMI (Peri et al., 2000). Heart myocytes experience constant physical stress. Whether such a stress is associated with PTX3 constitutive expression is not clear. PTX3 protein expression was shown to be increased in murine cardiomyocytes after transverse aortic constriction and H_2_O_2_ (Suzuki et al., 2003).

#### Endothelial cells

In atherosclerosis, high-density lipoprotein (HDL) induces the expression of PTX3 by activating a PI3K/Akt-dependent pathway in endothelial cells. Here PTX3 is suggested to manifest an anti-inflammatory and protective function (Norata et al., 2008). PTX3 is also induced by Lysophosphatidic acid (LPA), the lipid component of oxidized low-density lipoproteins (oxLDL) through the activation of NF-kB and is suggested to have a proatherogenic function (Gustin et al., 2008). These contradictory observations advocate the need of a unifying study to unveil the exact role of PTX3 in the development or resolution of atherosclerosis.

#### Epithelial cells

Alveolar epithelial cells (ECs) produce PTX3 upon mechanical stretch *in vitro* (Wu et al., 2009). LPS and TNF also induce production of PTX3 in ECs (Han et al., 2005). Human proximal renal tubular ECs constitutively express PTX3 mRNA, which is further upregulated by IL-1β, TNF, IL-17, and CD40L. IL-6 and IL-4 have no effect on PTX3 expression whereas GM-CSF was shown to diminish the effect of IL-1β (Nauta et al., 2005).

#### Chondrocytes

Enhanced expression of PTX3 was observed in synoviocytes and in synovial fluid from patients with rheumatoid arthritis (RA). IL-1β and oncostatin M (OSM) synergistically induce PTX3 expression in chondrocytes of the synovium (Barksby et al., 2006; Andreas et al., 2009) but whether or not PTX3 is involved in cartilage repair or degradation is largely unexplored. Expression of PTX3 in RA synoviocyte is however unaffected by TNF or IL-1β but is down regulated by TGF-β and IFN gamma (Luchetti et al., 2000).

#### Brain cells

Although PTX3 and neuronal pentraxins belongs to the same family, expression of former is generally not observed in the brain. It is, however induced by inflammatory stimuli such as LPS, TNF, and IL-1 in granule cells, presumptive glial cells in the white matter (corpus callosum, fimbria), meningeal pia mater, and dentate gyrus hilus (Polentarutti et al., 2000; Ravizza et al., 2001).

### PTX3 in lung diseases

The level of circulating PTX3 is low in healthy human condition (<2 ng/ml), but a rapid increase is observed in inflammatory conditions starting from very early stages. Due to its precocious appearance in various clinical conditions, research has focused on investigating whether PTX3 can be used as an index of systemic inflammatory activation. Another major reason suggestive of its suitability as a marker of severity of diseases like tuberculosis particularly in parasite endemic regions is the fact that its plasma level is unaffected by helminthic infections. There are several studies described in following sections, which demonstrate association between different diseased states and higher levels of PTX3.

#### Chronic obstructive pulmonary disorder

Chronic obstructive pulmonary disorder (COPD) is characterized by an irreversible airflow limitation that is associated with an abnormal inflammatory response in the lungs. The status of PTX3 in COPD patients is controversial. According to Van Pottelberge et al. (2012), no difference was observed in the levels of PTX3 in serum, sputum, pulmonary arteries, and alveolar space of COPD patients compared to healthy donors. However they claimed reduction in PTX3 positivity in lung sections obtained from moderate and severe COPD patients as compared to mild patients and healthy subjects (Van Pottelberge et al., 2012). Delneste and coworkers showed an increase in PTX3 level in serum and sputum of COPD patients compared to healthy people (Hamon et al., 2013). However, further investigation is required to determine whether this discrepancy in observation is due to the proteolytic cleavage of PTX3 in subjects participating in the former study or to the subjects’ demographic characteristics. Among various causative agents, cigarette smoke (CS) is a major cause of chronic COPD (Pauwels et al., 2010). Subacute and chronic exposure to CS augments PTX3 levels in the lung tissue of a murine COPD model, particularly in the pulmonary veins and venules. This upregulation was found to be dependent on the IL-1 pathway. Further, a critical role of PTX3 in the regulation of COPD-induced pulmonary inflammation, emphysema, and body weight changes was ruled out in this study because no significant difference was observed between CS exposed PTX3 knock-out (KO) and WT mice (Pauwels et al., 2010).

#### Asthma

Pentraxin 3 expression is also found to be associated with the pathology of asthma. In human biopsy samples collected from healthy and asthmatic subjects, our lab has found an enhanced expression of PTX3 in airway epithelium, infiltrating inflammatory cells, and airway smooth muscle bundles of asthmatic samples compared to their healthy counterparts. Interestingly, we did not observe any difference in PTX3 positivity between the lung sections from mild, moderate, and severe asthmatic. Studies examining the role of potential proteolytic cleavage of PTX3 would provide detailed insight into the regulation of PTX3 expression in asthma. We have also investigated the production of PTX3 in primary human airway smooth muscle cells (HASMCs) and ECs *in vitro* and found that HASMCs produce greater PTX3 as compared to ECs. Further, TNF and IL-1β were found to significantly upregulate its expression in HASMCs, but the effect of Th2, Th1, and Th17 cytokines was found to be negligible. PTX3 induces the production of eotaxin-1/CCL-11. Further, stimulation of HASMCs with PTX3 is found to inhibit fibroblast growth factor-2 (FGF-2) mediated migration (Zhang et al., 2012).

#### Lung carcinoma

Initially, PTX3 was suggested to be useful only as a marker of lung carcinoma based on studies performed on lung cancer cell lines (Planque et al., 2009) but more recent studies have determined that PTX3 could be used as a serum biomarker for the diagnosis and prognosis of lung carcinoma. Its suitability is overwhelming due to its ability to differentiate between cancer patients and non-cancer patients who are at higher risk of developing lung cancer (Diamandis et al., 2011).

#### Acute lung injury

Acute lung injury (ALI) and Acute Respiratory Distress Syndrome (ARDS) ALI/ARDS is characterized by injury associated with activation of the innate immune system in lungs. PTX3 is widely accepted as a marker of ALI because it is found in patients diagnosed with ALI within 24 h (He et al., 2007) and is closely associated with the severity of the disease. Concurrent infiltration of neutrophils, enhanced nitric oxide production, augmented expression, and function of tissue factor (TF) in lungs was observed in this pathological state (Lee et al., 1990, 1993, 1994). Consequently, it was suggested that a high level of PTX3 activates the local innate immune system, which was thought to serve a *protective role* against insults to which the lung tissue is exposed. LPS-induced ALI was investigated in PTX3 KO mice and the authors found that PTX3 KO mice were more susceptible to tissue damage as a result to LPS exposure compared to wild type mice (Okutani et al., 2007). In addition, PTX3 knock-in mice were observed to be better protected from LPS-induced endotoxemia (Dias et al., 2001). In an ALI model, PTX3 levels and the disease severity were found to be regulated by TF and TF, in turn, is induced by PTX3 (Han et al., 2011). Additional detailing of mechanisms in PTX3 KO mice other than the involvement of TF, which rendered them susceptible to ALI would shed more light on the role of PTX3.

### PTX3 and Lung infections

#### Pseudomonas aeruginosa infection

*Pseudomonas aeruginosa* is one of the prominent bacteria colonizing the lungs and causing chronic lung infections observed in Cystic Fibrosis (CF) patients. In humans, the PTX3 level increases in the serum of CF patients compared to healthy subjects (Hamon et al., 2013). Colonization of *P. aeruginosa* in CF patients is found to be positively associated with two intronic SNPs (rs1840680 and rs2305619) and one exonic SNP (rs3816527) in the PTX3 gene (Chiarini et al., 2010). In mouse, *protective role* of PTX3 was observed, as PTX3 KO mice are more susceptible to *P. aeruginosa* infection. Recombinant PTX3 facilitates the clearance of this pathogen by promoting an appropriate immune response in the lungs of PTX3 KO mice. This effect was demonstrated to be dependent on C3 and Fcγ and independent of C1q (Moalli et al., 2011). The mechanism by which this protective role is mediated through the complement system is described elsewhere in this review. Intraperitoneally administered PTX3 decreased the concentration of inflammatory mediators such as IL-1ββ, IL-17, CCL-2/MCP-1, CXCL1/KC, and CXCL2/MIP-2 in *P. aeroginosa* infected CF mice as compared to untreated CF mice. PTX3 treatment also decreased the infiltration of neutrophils and increased the percentage of monocytes in bronchoalveolar lavage fluid (BALF) from CF mice. Improvement in vascular leakage was also observed in CF mice upon PTX3 treatment (Paroni et al., 2012).

#### Aspergillosis

*Aspergillus fumigatus* is an opportunistic pathogen mainly infecting immunodeficient patients. In Invasive Pulmonary Aspergillosis (IPA), conidia of *A. fumigatus* induce the PTX3 secretion in the lungs which in turn recognizes and binds to the galactomannan moieties on the conidia and facilitates its phagocytosis by macrophages (Garlanda et al., 2002). Association of PTX3 with C3b and the resulting activation of C11b through FcγR are also involved in this process (Moalli et al., 2010). Additionally, PTX3 is also related to the amplification of the antifungal response by inducing the release of MCP-1/CCL-2 in mononuclear phagocytes thus promoting homing of monocytes to the lung tissue to aid in clearing the pathogen (Garlanda et al., 2002). PTX3 also activates the lectin complement pathway by binding to ficolin-L-bound *Aspergillus* conidia in an attempt to clear the infection (Moalli et al., 2010). Another mode of pathogen clearance is by the activation of DCs and subsequent induction of a Th1 response (Garlanda et al., 2002). In PTX3 KO mice, susceptibility to IPA was associated with increased levels of IL-4 (a Th2 cytokine) and decreased amounts of IFN gamma and IL-2 (Th1 cytokine). Pulmonary *A. fumigatus* infection is a major concern in immuno-compromised patients, particularly in bone marrow transplant patients and PTX3 allows a rapid recovery of myeloid and lymphoid cells into the lung tissue, which helps to accelerate the reconstitution of the patients’ immunity (Gaziano et al., 2004). Since cytomegalovirus (CMV) infections can also result in such an immuno-compromised state, PTX3 can resolve super infection by *A. fumigatus* even in a CMV-infected state (Bozza et al., 2006).

Pentraxin 3 shows differential binding patterns. It does not bind with hyphae but rather it binds only with the *A. fumigatus*, *A. flavus*, and *A. niger* conidia. However, interaction between PTX3 and fungal strains other than these such as *Candida albicans* is not observed (Garlanda et al., 2002). Thus direct action on *C. albicans* is not possible, but PTX3 can resolve *Candida* infection through binding with mannose binding lectin (MBL) (Table 1) and the subsequent recruitment of C1q and activation of classical complement cascade (Ma et al., 2009). The precise mechanism of *C. albicans* clearance from the lungs by PTX3 requires further investigation.

#### Tuberculosis

Plasma level of PTX3 is found to be correlated with the clinical severity of tuberculosis and therefore is seen as an appropriate indicator of the disease stage (Azzurri et al., 2005). PTX3 level declines with the success of therapeutic treatment against tuberculosis but increases again when treatment fails. This is also suggestive of the suitability of PTX3 as a tool to follow up the efficacy of treatment (Azzurri et al., 2005). Analysis of lung samples from human subjects determined that exposure to BCG vaccine leads to an increase in level of PTX3 (Aranday Cortes et al., 2010). This suggests that expression of PTX3 could be a protective mechanism and is not just a marker of the diseased state. One mechanism to confer immuno-protection may be mediated by monocytes that are induced to express PTX3 by the mycobacterial component (Mycobacterial lipoarabinomannan, LAM) (Vouret-Craviari et al., 1997). In humans, susceptibility to *Mycobacterium* tuberculi is also found to be positively associated with two intronic SNPs (rs1840680 and rs2305619) in PTX3 gene (Olesen et al., 2007). However some unanswered questions require further investigation, particularly, the mechanism of action of PTX3 on monocytes and involvement of other cell types in this process. It would also be worthwhile to determine any additional role for PTX3 plays additional roles in context with tuberculosis and its associated outcomes in the lungs.

#### Pneumonia

*Klebsiella pneumoniae* causes acute pulmonary infection in immuno-compromised subjects and results in pneumonia. Like other bacterial infections, PTX3 levels are found to be associated with the disease progression. A study by Soares et al. (2006) and Dias et al. (2001) determined that in PTX3 transgenic mice, high inoculum of bacteria induces overt expression of PTX3, and culminates in greater lethality. Very high levels of PTX3 inhibit neutrophil influx in the lungs due to inhibition of P-selectin (Deban et al., 2010) and enhanced NO production and iNOS expression (Soares et al., 2006). As a result of this, production of TNF was found inhibited, resulting in greater bacteria count in lung tissue (Soares et al., 2006). Since TNF induces PTX3 expression, one explanation for reduced production of TNF in this infection model could be a feedback mechanism to regulate PTX3 levels and exacerbations that may result from its very high levels. However, when PTX3 transgenic mice were infected with a smaller inoculum, protective effects of PTX3 were conferred and was attributed to enhanced TNF production, increased neutrophil infiltration to lungs and decreased bacterial load in lungs and blood (Soares et al., 2006). It is quite possible that such a protective effect is initiated by a mechanism that involves PTX3 induction by [Outer membrane protein A (OmpA) of *K. pneumoniae*] through TLR2, which in turn binds OmpA (Table 1) and amplifies the activation of complement cascade and promotes the opsonophagocytosis of OmpA containing bacteria (Jeannin et al., 2005; Cotena et al., 2007). In humans, a positive correlation is observed between plasma levels of PTX3 and the severity of Community acquired pneumonia (CAP) and that PTX3 concentration decreased upon treatment with antibiotics. The PTX3 level was also found to be significantly correlated with the length of the hospital stay for CAP patients (Kao et al., 2012).

#### Cytomegalovirus infection

Cytomegalovirus is a herpes virus, present in the majority of the general population. It exists in latent form even after the infection is treated and may reactivate in an immunocompromised state (Forbes, 1989). PTX3 not only provides protection at very early stages of CMV infection, but also suppresses reactivation (Bozza et al., 2006). PTX3 mediates its action by binding to hemagglutinin (Table 1) on the surface of the virus and blocks its entry into host cells, particularly DCs (Bozza et al., 2006). It can also induce antiviral immunity through the activation of mDCs and subsequent up regulation of appropriate T cells functions (Bozza et al., 2006). Virus-bound PTX3 acts through TLR2, 3, and 4 and induces the production of IFN gamma and IL-12 through the transcription factor IRF3 in CD11b^+^ DCs C57BL/6 mice (Bozza et al., 2006). mDCs themselves also secrete PTX3 upon exposure to viral particles and maintains the loop of infection followed by immuno-protection. Exogenous PTX3 is a promising potential therapeutic agent against CMV infection, both alone (Bozza et al., 2006) and in combination with Thymosin alpha-1 (Patent: use of thymosin alpha-1, alone and in combination with PTX3 or Ganciclovir, for the treatment of cytomegalovirus infection).

#### Influenza

The Influenza virus presents another serious challenge to the respiratory tract because of its ability to mutate and create virulent variants as a mechanism to evade the host immune system. Influenza viruses are RNA orthomyxoviruses, which infect ECs of the respiratory tract (Matrosovich et al., 2004). A detailed study by Reading et al. (2008) showed that like CMV, a sialic moiety of PTX3 engages hemagglutinin of the influenza virus and hinders attachment of the virus to host ECs. Inhibition of viral neuraminidase glycoprotein by PTX3 could be an additional strategy to limit viral infection as it inhibits the release of newly formed viral particles from the infected host cells (Kilbourne et al., 1968). PTX3 also facilitates opsonization and clearance of infected cells. PTX3 KO mice are more prone to influenza virus infection but attained resistance when treated with exogenous PTX3 (Reading et al., 2008).

#### Severe acute respiratory syndrome

Severe Acute Respiratory Syndrome (SARS) is caused by SARS coronaviruses (SRAS-CoV) (Rota et al., 2003). The role of PTX3 is discussed with respect to murine hepatitis virus (MHV) which is a group 2 coronavirus that is closely related to its human counterpart SARS-CoV (Rota et al., 2003; Han et al., 2012). MHV-1 infection results in ALI in mice, similar to airways damage observed in the lungs of SARS patients (De Albuquerque et al., 2006). MHV-1 airway infection induces PTX3 expression in the lungs. PTX3 bound to MHV-1, reduced its infectivity and accelerated viral clearance. Consequently, PTX3 KO mice showed greater pulmonary damage as compared to their WT counterparts and the animals that were treated with exogenous PTX3. In PTX3 KO mice, an early influx of neutrophils and macrophages into the lungs is found to exaggerate lung injury due to MHV-1 infection. Other inflammatory determinants such as IL-6, MCP-1, and MIP-1b were found to be enhanced in the PTX3 KO condition. However, production of these inflammatory cytokines was reduced upon PTX3 treatment, resulting in protection from airway damage (Han et al., 2012).

As a matter of caution, PTX3 is not a general mechanism to guard against all pathogenic infections. Infection due to *Listeria monocytogenes* and *Salmonella typhimurium* is neither controlled nor exaggerated by PTX3 directly or indirectly. Similarly, PTX3 is unable to bind and mediate protection against some variants of Influenza virus such as A/PR8/34 (HINI), H3N3, and type B influenza viruses (Reading et al., 2008).

### Mechanism of action

Pentraxin 3 exerts diverse functions to provide immuno-protection in multiple ways. It binds to the surface of pathogens and apoptotic inflammatory cells and promotes their opsonization and clearance early in the infection process. This role is important to avoid inducing a deleterious hyper-inflammatory state that could arise due to activation of the adaptive arm of the immune response. Notably, although the N-terminal domain of PTX3 is suggested to be required for pathogen binding, the full-length protein is required for opsonizatio (Moalli et al., 2010). Also PTX3 when bound to pathogenic components activates DCs, which is instrumental in initiating an appropriate T cell response. Another mechanism by which PTX3 initiates the thread of innate immunity is by activating the complement cascade (Bozza et al., 2006).

#### Complement pathway activation

A prototypical paradigm by which PTX3 activates the classical complement pathway is by interacting with C1q (Table 1). PTX3 binds to the globular head of immobilized C1q through its C-terminal domain, subsequently triggering the activation of the downstream cascade. Glycosidic patterns on PTX3 significantly contribute to its interaction with C1q (Inforzato et al., 2006). Of note, when C1q is in solution, PTX3 inhibits the activation of complement pathway by blocking C1q interaction with immunoglobulins or other agents (Baruah et al., 2006).

Eventually, the ability of PTX3 to resolve *Aspergillus* infection in C1q KO mice but not in C3 KO mice indicated that PTX3 might also interact with other members of the complement family to mediate its function. PTX3 amplifies C3b-mediated opsonization and phagocytosis of *Aspergillus* conidia through inside-out activation of CD11b in macrophages (Moalli et al., 2010). Conidia-bound PTX3 binds to Factor H (Table 1) and promotes C3-mediated activation of the alternate pathway (C3 is also deposited on conidia) and killing of the pathogen. A similar mechanism is employed to remove apoptotic cells which can be a potential cause of unwanted inflammation (Deban et al., 2008). Factor H binds to PTX3 at two sites: the PTX3 N-terminus acts as the primary binding site and the glycosylated pentraxins domain acts as the secondary binding site (Deban et al., 2008). While investigating the role of PTX3 in the disposal of *Aspergillus* conidia, Moalli et al. (2010) found that factor B of the alternate pathway is also necessary for its activity.

Pathogens attempt to evade the immune system so as to infect the host, in turn, activates multiple immune mechanisms to fight against the infection. The work of Moalli et al. (2010) implicated the role of all complement pathways in conidia opsonization. The association of PTX3 with other complement components further strengthens the suggestion. Along the same line, PTX3 was also found to activate the lectin complement pathway, by interacting with ficolins through their fibrinogen-like domain (Table 1) (Martin et al., 2009). Ficolins recognize carbohydrate moieties on pathogens and dying cells and provide immuno-protection by activating the lectin complement pathway and prime the adaptive immune response (Endo et al., 2011). Besides binding to ficolin-L, PTX3 enhances complement activation resulting from ficolin-L interaction with *Aspergillus* (Ma et al., 2009, 2011). However the interaction between ficolin-L and PTX3 is critically affected by polymorphism in ficolin-L gene causing a T236 amino acid change in the fibrinogen-like domain (Ma et al., 2009). Similarly, ficolin-M also interfaces with PTX3 (Ma et al., 2009). This may be of even greater significance in this review because ficolin-M is majorly produced and found in the lungs (Ma et al., 2009; Gout et al., 2011). The ficolin-M tetramer binds to four PTX3 molecules where a sialic moiety in an N-linked carbohydrate of the C-terminal domain of later was found to be involved (Gout et al., 2011).

### PTX3 receptor and ligands

Because PTX3 acts on immune cells, playing a critical role in driving innate immunity against pathogens, it is possible that PTX3 might act through some unknown receptor. A study investigating the binding properties of pentraxins with FcyRs determined that PTX3 could recognize FcyRs (Lu et al., 2008) (Table 1). The functional significance of such an interaction was later established in an *Aspergillus* infection model (Moalli et al., 2010). The protective effect of PTX3 against *Aspergillus* was found to be diminished in the absence of FcγR. This interaction was suggested to activate CD11b to induce opsonophagocytosis of the pathogen (Moalli et al., 2010). With the diverse functions mediated by PTX3 at the cellular level, it is likely that PTX3 may interact with additional unknown receptors, which could be the subject of further investigation.

Pentraxin 3 binds to FGF-2 (Rusnati et al., 2004; Camozzi et al., 2006) and fibroblast growth factor-8b (FGF-8b) (Leali et al., 2011) (Table 1) and affects neovascularization by mediating anti-angiogenic and anti-restenotic activity. FGF-2 induces proliferation in vascular smooth muscle cells and endothelial cells, which is inhibited by PTX3 (Rusnati et al., 2004; Camozzi et al., 2005). PTX3 inhibits FGF-2 functions by physically interacting through its N-terminal domain (Camozzi et al., 2006). TSG-6 also interacts with PTX3 through the same domain and as a result, it competes with FGF-2, thus abolishes the inhibitory effect of PTX3 on FGF-2-mediated angiogenesis (Leali et al., 2012). The association between TSG-6 and PTX3 is extremely critical in orchestrating ECM in cumulus oophorus, which is important for female fertility (Salustri et al., 2004). Thus because PTX3 and TSG-6 were found to be co-regulated in monocytes, macrophages and myeloid DCs, their association was suggested to contribute to ECM remodeling during inflammation (Maina et al., 2009).

## Concluding Remarks

Pentraxin 3, a unique member of the long pentraxins family, plays an indispensable role in regulating our immune system against pathogens, which are involved in several pulmonary pathologies. Multiple mechanisms to recognize pathogens and to coordinate with the activation of humoral and cell-mediated immunity might explain why it is evolutionarily conserved from non-vertebrates to complex and highly evolved vertebrates. Several disease and infection models have been extremely useful in understanding the role of PTX3 in normal and immuno-compromised disease states. However, detailed and comprehensive investigations unveiling the mechanisms by which PTX3 may modulate the immune system are certainly needed. PTX3 is an essential factor in determining female fertility, as it is instrumental in matrix deposition in cumulus oophorus. Characterization of its role in other physiological processes also requires further research.

## Conflict of Interest Statement

The authors declare that the research was conducted in the absence of any commercial or financial relationships that could be construed as a potential conflict of interest.
